# Risk Factors and Post-Resection Independent Predictive Score for the Recurrence of Hepatitis B-Related Hepatocellular Carcinoma

**DOI:** 10.1371/journal.pone.0148493

**Published:** 2016-02-22

**Authors:** Ivan Fan-Ngai Hung, Danny Ka-Ho Wong, Ronnie Tung-Ping Poon, Daniel Yee-Tak Fong, Ada Hang-Wai Chui, Wai-Kay Seto, James Yan-Yue Fung, Albert Chi-Yan Chan, John Chi-Hang Yuen, Randal Tiu, Olivia Choi, Ching-Lung Lai, Man-Fung Yuen

**Affiliations:** 1 Department of Medicine, the University of Hong Kong, Queen Mary Hospital, Hong Kong, China; 2 State Key Laboratory for Liver Research, the University of Hong Kong, Queen Mary Hospital, Hong Kong, China; 3 Department of Surgery, the University of Hong Kong, Queen Mary Hospital, Hong Kong, China; 4 School of Nursing Studies, the University of Hong Kong, Queen Mary Hospital, Hong Kong, China; 5 Quality & Safety Division, New Territories West Cluster, Hospital Authority, Hong Kong, China; 6 Department of Clinical Oncology, the University of Hong Kong, Queen Mary Hospital, Hong Kong, China; Kaohsiung Medical University Hospital, Kaohsiung Medical University, TAIWAN

## Abstract

**Background:**

Independent risk factors associated with hepatitis B (HBV)-related hepatocellular carcinoma (HCC) after resection remains unknown. An accurate risk score for HCC recurrence is lacking.

**Methods:**

We prospectively followed up 200 patients who underwent liver resection for HBV-related HCC for at least 2 years. Demographic, biochemical, tumor, virological and anti-viral treatment factors were analyzed to identify independent risk factors associated with recurrence after resection and a risk score for HCC recurrence formulated.

**Results:**

Two hundred patients (80% male) who underwent liver resection for HBV-related HCC were recruited. The median time of recurrence was 184 weeks (IQR 52–207 weeks) for the entire cohort and 100 patients (50%) developed HCC recurrence. Stepwise Cox regression analysis identified that one-month post resection HBV DNA >20,000 IU/mL (p = 0.019; relative risk (RR) 1.67; 95% confidence interval (C.I.): 1.09–2.57), the presence of lymphovascular permeation (p<0.001; RR 2.69; 95% C.I.: 1.75–4.12), microsatellite lesions (p<0.001; RR 2.86; 95% C.I.: 1.82–4.51), and AFP >100ng/mL before resection (p = 0.021; RR 1.63; 95% C.I.: 1.08–2.47) were independently associated with HCC recurrence. Antiviral treatment before resection (p = 0.024; RR 0.1; 95% C.I.: 0.01–0.74) was independently associated with reduced risk of HCC recurrence. A post-resection independent predictive score (PRIPS) was derived and validated with sensitivity of 75.3% and 60.6% and specificity of 55.7% and 79.2%, to predict the 1- and 3-year risks for the HCC recurrence respectively with the hazard ratio of 2.71 (95% C.I.: 2.12–3.48; p<0.001). The AUC for the 1- and 3-year prediction were 0.675 (95% C.I.: 0.6–0.78) and 0.746 (95% C.I.: 0.69–0.82) respectively.

**Conclusion:**

Several tumor, virological and biochemical factors were associated with a higher cumulative risk of HCC recurrence after resection. PRIPS was derived for more accurate risk assessment. Regardless of the HBV DNA level, antiviral treatment should be given to patients before resection to reduce the risk of recurrence.

## Introduction

Hepatocellular carcinoma (HCC) is the sixth most common cancer worldwide [1]. According to cancer statistics released by the World Health Organization in 2008, HCC ranked the third in the cancer mortality globally [2]. 78% of liver cancer is caused by either chronic hepatitis B (CHB) or C infection [3]. CHB increases the risk by more than 20-fold. In Hong Kong, the prevalence of CHB infection is approximately 8% [4]. Patients with CHB are estimated to experience HCC at a rate of 0.5% per year [5]. This rate increases to 2–3% per year in CHB patients who already had cirrhosis [6], highlighting the importance and impact of hepatitis B virus (HBV)-related HCC in the morbidity and mortality in high CHB prevalence population.

Investigators have identified several important viral factors, including high HBV viral load, genotype C, core promoter mutation, pre-S deletions, and presence of T1653 and V1753 mutations to be associated with increased risk of developing HCC in CHB patients [7–10]. For patients who develop HCC, 20–30% are candidates for curative resection [11]. Nevertheless, the intrahepatic recurrence rate after tumor resection is estimated to be >50% after 3 years [12].

In a previous relatively small-sized study, we have identified that age >60 years, tumor size >5cm, α-fetoprotein (AFP) level >1000ng/ml and HBV viral load >2,000 IU/mL after tumor resection, to be associated with increased cumulative risk of HCC recurrence [13]. We have also completed a large-scale study using a statistical method to formulate a predictive score to estimate the chance of occurrence of HCC as a time-dependent event during prospective follow-up [14]. Based on these works, we conducted a large scale prospective-retrospective study in patients who underwent resection for HBV-related HCC to identify independent risk factors associated with recurrence of HCC and derived a novel post-resection independent predictive score (PRIPS) for the development of recurrence. This novel risk score PRIPS would be useful to predict the chance of recurrence of the HCC after resection and hence may provide important guidance for subsequent surveillance strategy for HCC recurrence, and the development of novel therapeutic strategies in the prevention of recurrence.

## Patients and Methods

### Study Design and Ethics

This study was designed in 2009 by the Departments of Medicine and Surgery, the University of Hong Kong, Queen Mary Hospital. The study protocol conformed to the ethical guidelines of the 1975 Declaration of Helsinki and was approved by the institutional review board of the University of Hong Kong/ Hospital Authority, Hong Kong (Certificate number: UW 08–157). No written or oral consent was obtained from the participants. The reason was that it was not an interventional study and all data was analyzed anonymously. The IRB committee has approved this consent procedure.

### Patient’s selection

We prospectively recruited patients diagnosed to have HBV-related HCC who underwent curative tumor resection between July 2009 and June 2011, in the Department of Surgery, the University of Hong Kong, Queen Mary Hospital. All recruited patients were either at stage 0 or A, according to the Barcelona Clinic Liver Cancer Staging upon resection. In addition, we included the 72 patients who were recruited and followed up in the previous study [13]. Diagnosis of HBV-related HCC was based on imaging techniques including computerized tomography (CT), hepatic angiography, magnetic resonance imaging and AFP level. All patients had HCC confirmed by histological examination of the resected surgical specimens and HBV infection confirmed by positive hepatitis B surface antigen (HBsAg) in the serum. HCC recurrence is defined as development of new intrahepatic lesions at least 6 months after the first HCC resection. Patients with other concomitant liver diseases including hepatitis C or D viral infection, autoimmune hepatitis, Wilson’s disease, primary biliary cirrhosis, alcoholic liver disease, fatty liver (diagnosed by ultrasonography) and extrahepatic metastasis diagnosed before resection were excluded from the study. Patients who suffered mortality from other causes besides liver cause were censored at the last follow-up upon the time of death. Patients with other significant co-morbidities were not recruited. Patient’s history of antiviral treatment given before and within six months after resection was recorded.

### Preoperative Investigations

Baseline demographic data including patient’s sex and age at first tumor resection were recorded. Serum samples within 2 weeks before HCC resection were taken for liver function tests and AFP. Abnormal alanine transaminase (ALT) is defined as a value of above the upper limit of normal that is >53 IU/L for male and >37 IU/L for female, whereas abnormal AFP is defined as >20ng/mL. Model for end stage liver disease (MELD) score and Child-Pugh score for cirrhosis upon resection was calculated.

### Viral Factors

Viral markers including HBsAg, hepatitis B e antigen (HBeAg) and antibody to HBeAg (anti-HBe) were measured by the AxSYM immunoassay systems (Abbott Laboratories, Chicago, IL). Specific HBV mutations including precore mutations (G1896A), core promoter mutations (A1762T/G1764A), pre-S deletions, C1653T and T1753V mutations were determined by direct sequencing in which the detailed methodologies were described in previous studies [15,16]. HBV genotypes were determined by phylogenetic comparison of the pre-S/S sequences with reference HBV sequences in the NCBI GenBank. Serum samples were obtained at three time points: before, and at one and six months after surgery for quantification of serum HBV DNA, measured by the COBAS Taqman HBV Test (Roche Diagnostics, Branchburg, NJ) with a lower limit of detection of 20 IU/mL.

### Surgery and Tumor Factors

Surgery factors including blood loss during operation and techniques of hepatic resection (anatomical or non-anatomical resection) were recorded. The resected tumors with its surrounding liver were examined for its histopathological features. The tumor size, resection margin, histological grade, presence of microsatellite formation and vascular permeation in the resected tumor, together with the presence of cirrhosis in the non-tumorous liver were determined.

### Follow-up

All patients were followed up at three monthly intervals with liver function test, AFP levels, chest x-ray and helical contrast CT of the abdomen after resection to monitor for both intrahepatic and extrahepatic recurrence. All patients were follow-up for at least a period of 104 weeks after resection.

### Statistical analysis

Statistical analyses to identify risk factors for recurrence were performed using the SPSS 21.0 (IBM SPSS Statistics, IBM Corp, Somers, NY). Log-rank test was used to compare the cumulative risks of development of HCC recurrence. Multivariable stepwise Cox regression model was used to determine independent risk factors associated with HCC recurrence.

The PRIPS was then calculated as the weighted sum of those significant risk factors, with weights taken as the estimated coefficients in multivariable Cox regression model. Prediction accuracy of the risk score for 1- and 3-year HCC recurrence was assessed by the area under the curve (AUC) in a time-dependent Receiver Operating Characteristic curve (ROC) analysis [17]. The corresponding 95% confidence intervals (CI) were obtained by bootstrapping of size 1000 with confidence limits taken as the 2.5^th^ and 97.5^th^ percentiles of the bootstrap samples. Tentative optimal cutoff values for predicting 1- and 3-year HCC recurrence were obtained as those that maximized the Youden index, that was sensitivity and specificity—1, as derived from the time-dependent ROC analysis. At the optimal cutoff values, sensitivity, specificity, predictive values and likelihood ratios, as well as their corresponding 95% CIs were calculated. For practical reason, the final risk score was subtracted by the tentative optimal cutoff values such that the optimal cutoff values were 0, i.e. a risk score ≥0 predict HCC recurrence. Finally, we validated the optimal cutoff values by using the leave-one-out cross-validation method [18]. The time-dependent ROC and cross-validation analyses were performed in R 3.0.1 version [19].

## Results

Two hundred patients with resection for HBV-related HCC who fulfilled the inclusion criteria were recruited (Table 1). Seventy-two patients (36%) were recruited between June 2004 and December 2006 from the previous study and the remaining 128 patients were recruited between July 2009 and June 2011. The median age upon diagnosis of HCC was 56 years [interquartile range (IQR) 49–65)] and 160 patients (80%) were male. Thirty-one patients (15.5%) were HBeAg positive. The median follow-up time was 207 weeks (IQR 182–458 weeks) for patients with no recurrence. By the end of the study, 100 patients (50%) developed HCC recurrence, of which 53 patients died. For the entire cohort, the median time of recurrence was 184 weeks (3.5 years) (IQR 52–207 weeks) for the entire cohort of 200 patients. In comparison, no patients died in the non-tumor recurrence group (p<0.001) (Table 1). Five patients died of pneumonia, three patients died of stroke and 1 patient died of congestive heart failure. The remaining 44 patients died of HCC. None of the patients in the non-tumor recurrence group died.

**Table 1:** Virological and clinic-pathological factors of the 200 patients in cumulative recurrence of hepatocellular carcinoma after resection by log-rank test.

	Tumor Recurrence (n = 100)	No Tumor Recurrence (n = 100)	p Value
**Demographic Factors**			
Median age (IQR) years	56 (49–66)	56 (49.3–63)	0.26
Age >60 years	38%	34%	0.73
Male	79%	81%	0.77
**Biochemical Factors**			
AFP >100ng/mL	48%	35%	**0.01**
MELD score (IQR)	6.4 (4.95–8.20)	6.5 (5.1–8.28)	0.90
Child-Pugh score A	96%	96%	1.00
Cbild-Pugh score B	4%	4%	1.00
**Surgery Factors**			
Blood loss (L)	0.58 (0.2–1.2)	0.45 (0.15–1.0)	0.34
Anatomical resection	85%	89%	0.40
**Tumor Factors**			
BCLC stage 0	12%	14%	0.67
BCLC stage A	88%	86%	0.67
Tumor number = 1	92%	93%	0.72
Tumor number = 2	7%	5%	
Tumor number = 3	1%	2%	
Tumor size >3cm	72%	57%	**0.026**
Poor differentiated tumor	88%	62%	**<0.001**
Presence of microsatellite lesions	31%	6%	**<0.001**
Lymphovascular permeation	52%	19%	**<0.001**
Cirrhosis	60%	54%	0.47
**Viral Factors**			
Pre-resection HBV DNA >20,000 IU/mL	66%	44%	**0.002**
1 month post-resection HBV DNA >20,000 IU/mL	56%	34%	**0.002**
6 months post-resection HBV DNA >20,000Iu/mL	37%	23%	**0.031**
Pre-S deletion	18%	17%	0.93
Positive eAg	16%	15%	0.78
C1653T	23%	10%	**0.042**
T1753V	29%	25%	0.87
Genotype C	64%	50%	0.39
Presence of core promoter mutation	82%	60%	**0.032**
Presence of precore mutation	33%	31%	0.95
**Antiviral Treatment**			
Antiviral treatment before resection	1%	23%	**<0.001**
Antiviral treatment after resection	43%	49%	0.40
**Outcome**			
Death	53%	0%	**<0.001**

All 200 patients had HBV DNA available before surgery for sequence analysis. One hundred and seventy-three patients had sufficiently high HBV DNA levels for PCR amplification for analysis of genotyping, pre-S deletions, core promoter, precore, C1653T and T1753V mutations, in which 34.1% were genotype B and 65.9% were genotype C; 20.2% had pre-S deletions; 82.1% had core promoter mutations; 40% had precore mutations; 19.1% had C1653T mutations and 31.2% had T1753V mutations.

### Demographic Factors and HCC Recurrence

Patients with age >60 years upon diagnosis and gender were not associated with HCC recurrence (Table 1). Recurrence was strongly associated with death (p<0.001).

### Biochemical Factors and HCC Recurrence

AFP >100ng/mL (p = 0.01) at the time of resection was associated with a higher cumulative risk of developing HCC recurrence after resection (Table 1). There was no difference in the MELD and Child-Pugh scores upon resection between the two groups.

### Surgery Factors and HCC Recurrence

There was no difference in blood loss and resection techniques between the two groups (Table 1).

### Tumor Factors and HCC recurrence

Resected tumor size >3cm (p = 0.026); moderate or poor differentiated HCC (p<0.001), the presence of lymphovascular permeation (p<0.001) and the presence of microsatellite lesions (p<0.001) at the time of resection were associated with a higher cumulative risk of developing HCC recurrence after resection (Table 1). 114 (57%) patients were diagnosed to have underlying cirrhosis, which was not associated with recurrence. 26 (13%) patients have Barcelona-Clinic Liver Cancer (BCLC) stage 0 and 174 (87%) patients have BCLC stage A diseases respectively upon diagnosis. 185 patients (92.5%) have single lesion, 12 patients (6%) have two lesions and 3 patients (1.5%) have three lesions respectively. There was no difference in BCLC staging and number of tumors between the two groups.

### Virological Factors and HCC Recurrence

Despite only 31 patients (15.5%) were HBeAg positive, the median HBV DNA at the time of resection remained high at 5.4x10^4^ IU/mL (IQR 2.4x10^2^–1.1x10^5^ IU/mL). Patients with pre-resection HBV DNA >20,000 IU/mL (p = 0.002), 1 and 6 months post-resection HBV DNA >20,000 IU/mL (p = 0.001 and p = 0.02 respectively), the presence of core promoter (p = 0.032) and C1653T (p = 0.042) mutations was associated with a higher cumulative risk of developing HCC recurrence after resection (Table 1). Genotypes, pre-S deletion, precore and T1753V mutations were not associated with cumulative risk of developing HCC recurrence after resection.

### Antiviral Treatment and HCC Recurrence

Twenty-four and 92 patients received antiviral treatment before and within 6 months after resection respectively. Out of the 24 patients who received antiviral treatment before resection, 8 patients (33.3%) had HBV DNA >20,000 IU/mL before resection and 22 patients (91.7%) had undetectable HBV DNA at 6 months after resection. Only 1/24 patients (4.2%) and none of the 8 patients with HBV DNA >20,000 IU/mL developed HCC recurrence. Patients who received antiviral treatment before resection were associated with significantly lower cumulative risk of recurrence compared to patients with no anti-viral treatment (p<0.001) (Table 1).

For those patients who received antiviral treatment within 6 months after resection, 62/92 (67.4%) had HBV DNA >20000 IU/mL before resection, 36/92 (39.1%) had undetectable HBV DNA at 6 months after resection and 43/92 (46.7%) developed HCC recurrence.

### Independent Risk Factors for HCC Recurrence

**Fig 1:**
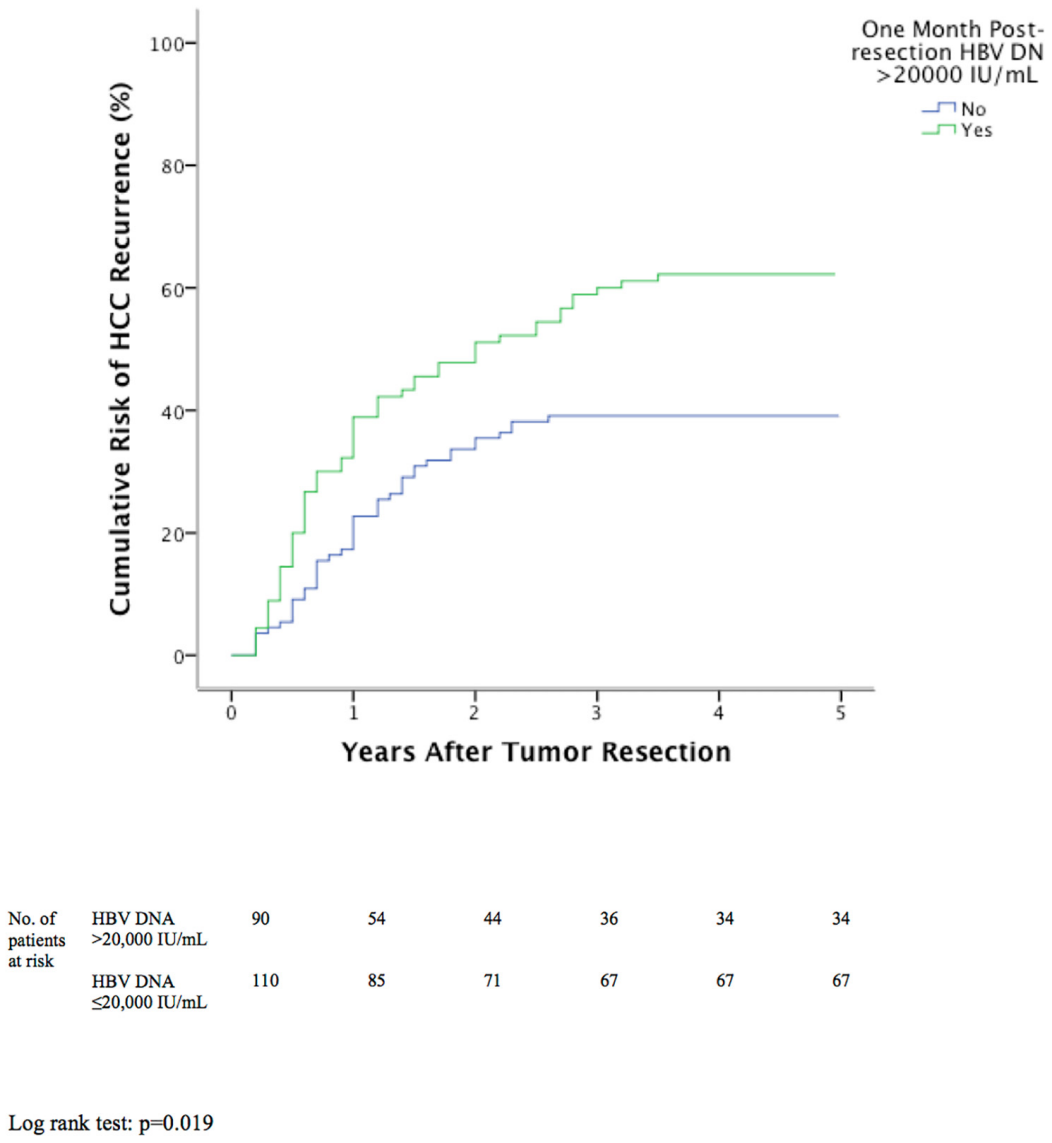
Cumulative tumor recurrence between patients with or without one month post-resection HBV DNA >20,000 IU/mL.

**Fig 2:**
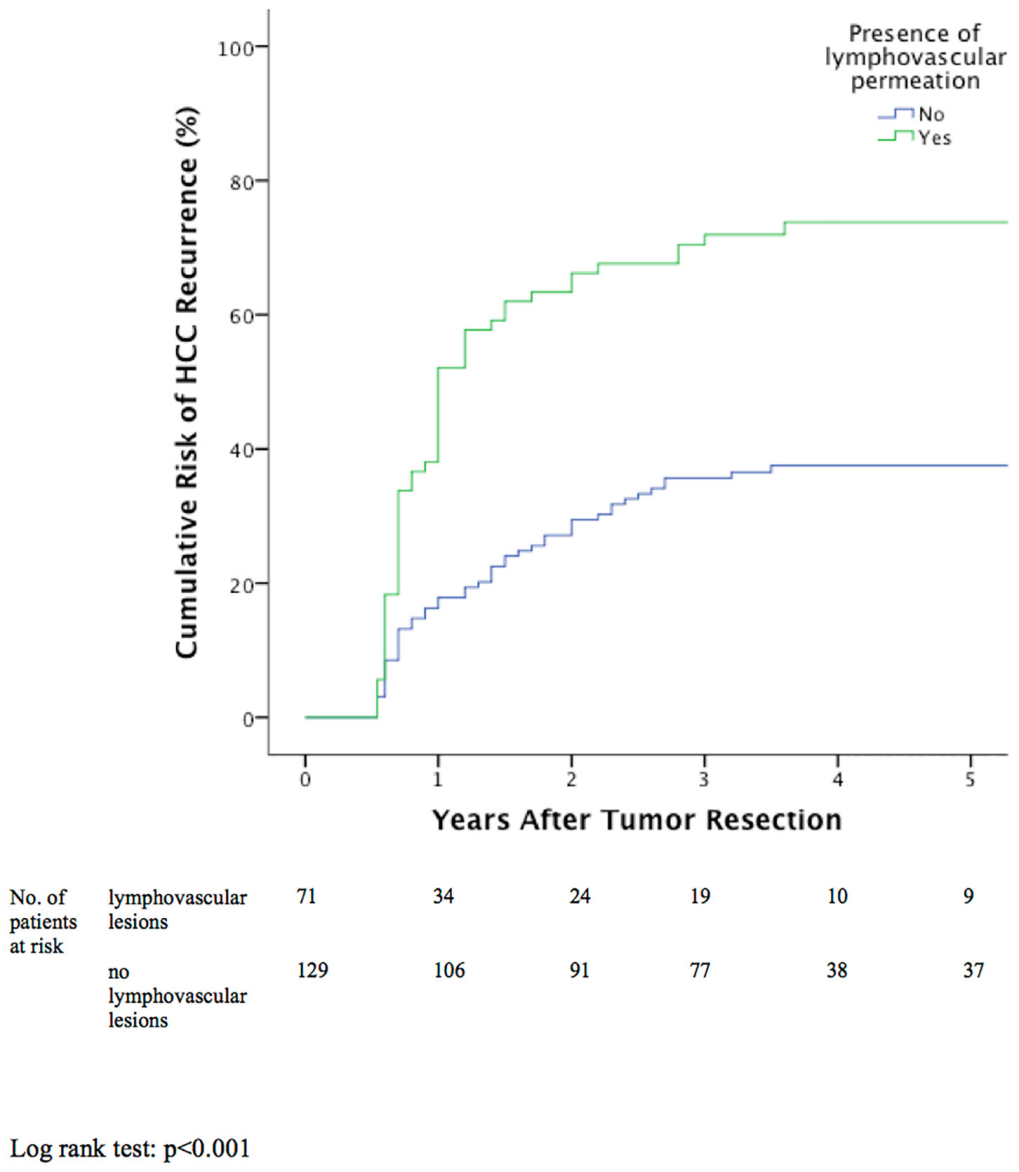
Cumulative tumor recurrence between patients with or without lymphovascular permeation.

**Fig 3:**
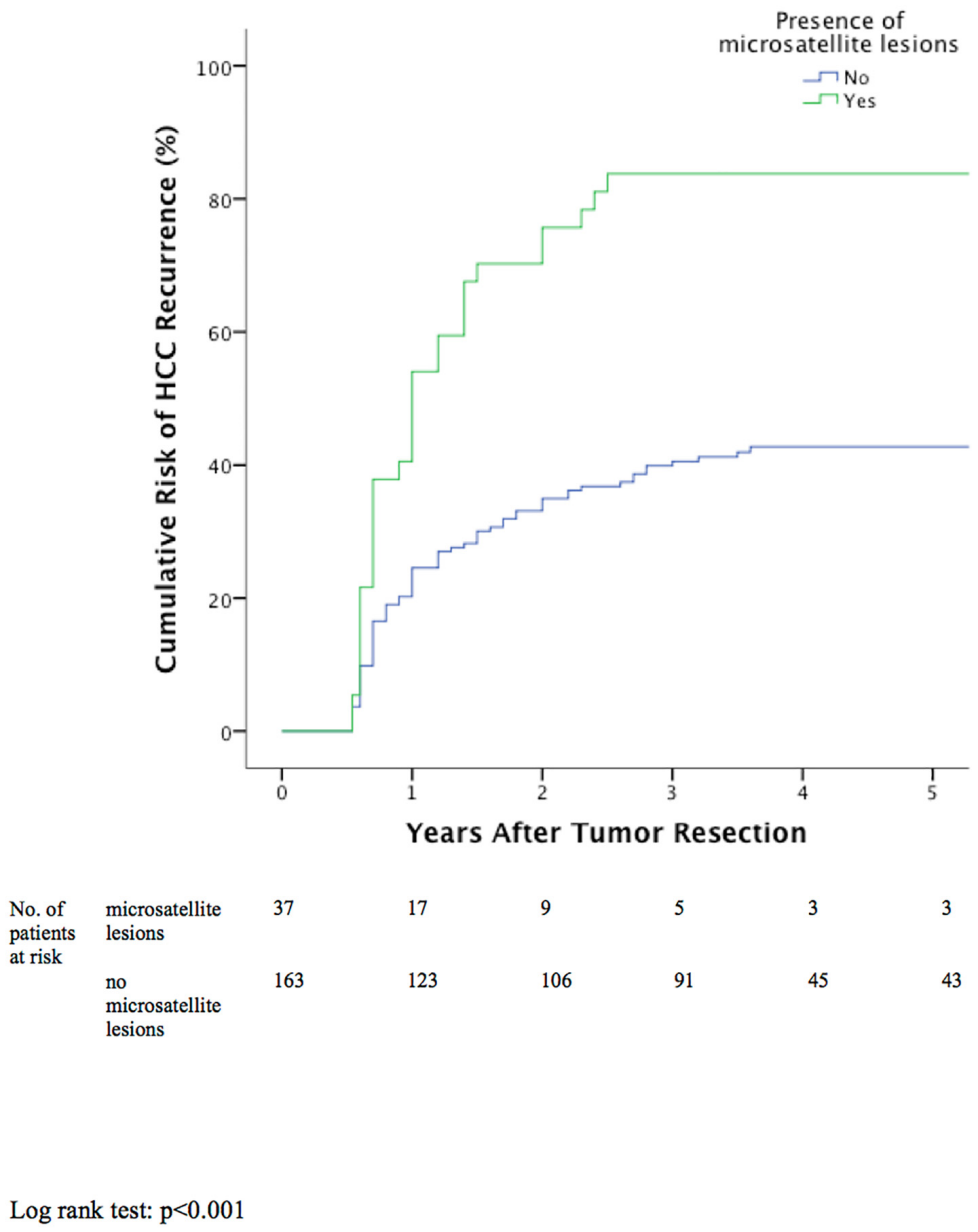
Cumulative tumor recurrence between patients with or without microsatellite lesions.

**Fig 4:**
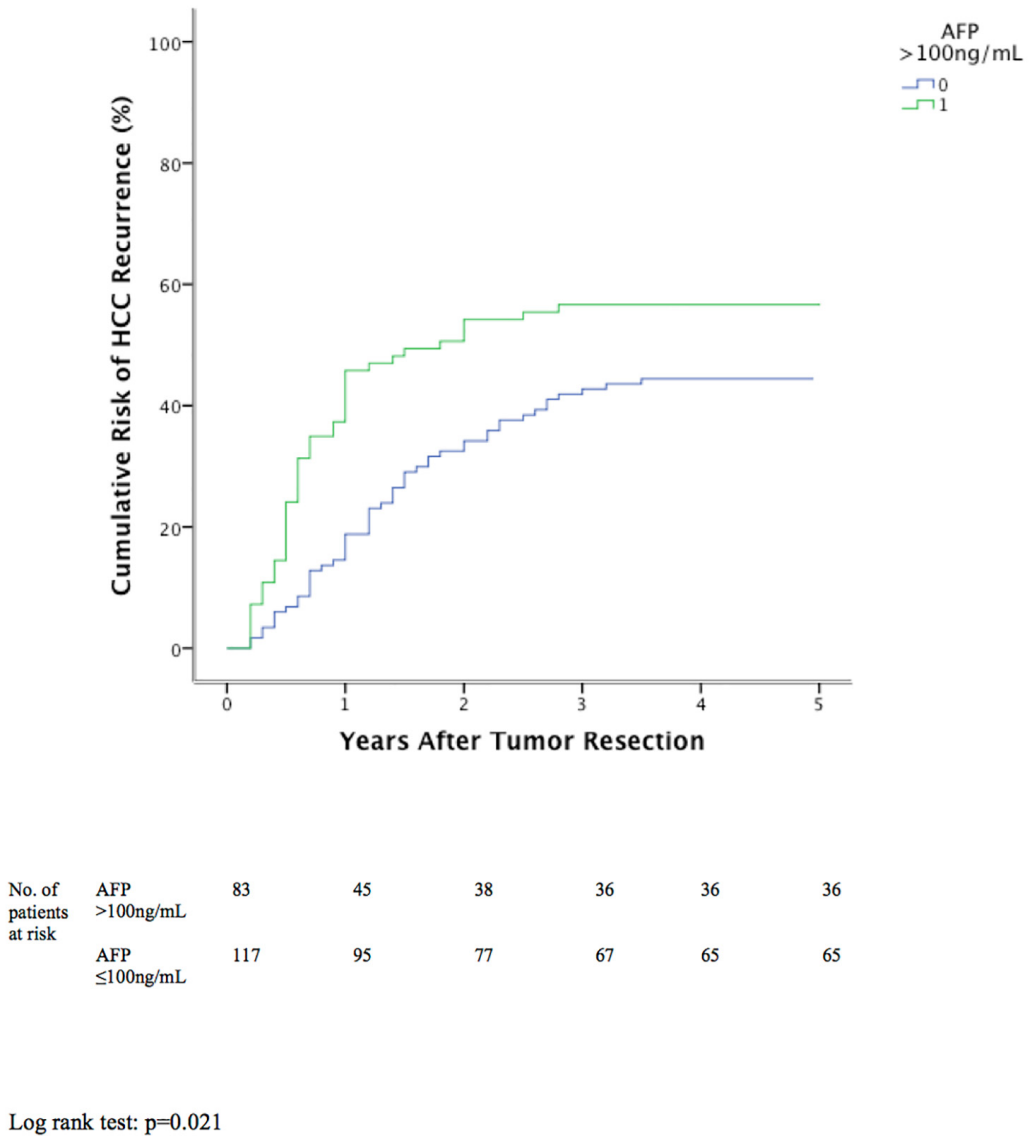
Cumulative tumor recurrence between patients with or without AFP >100ng/mL before resection.

**Fig 5:**
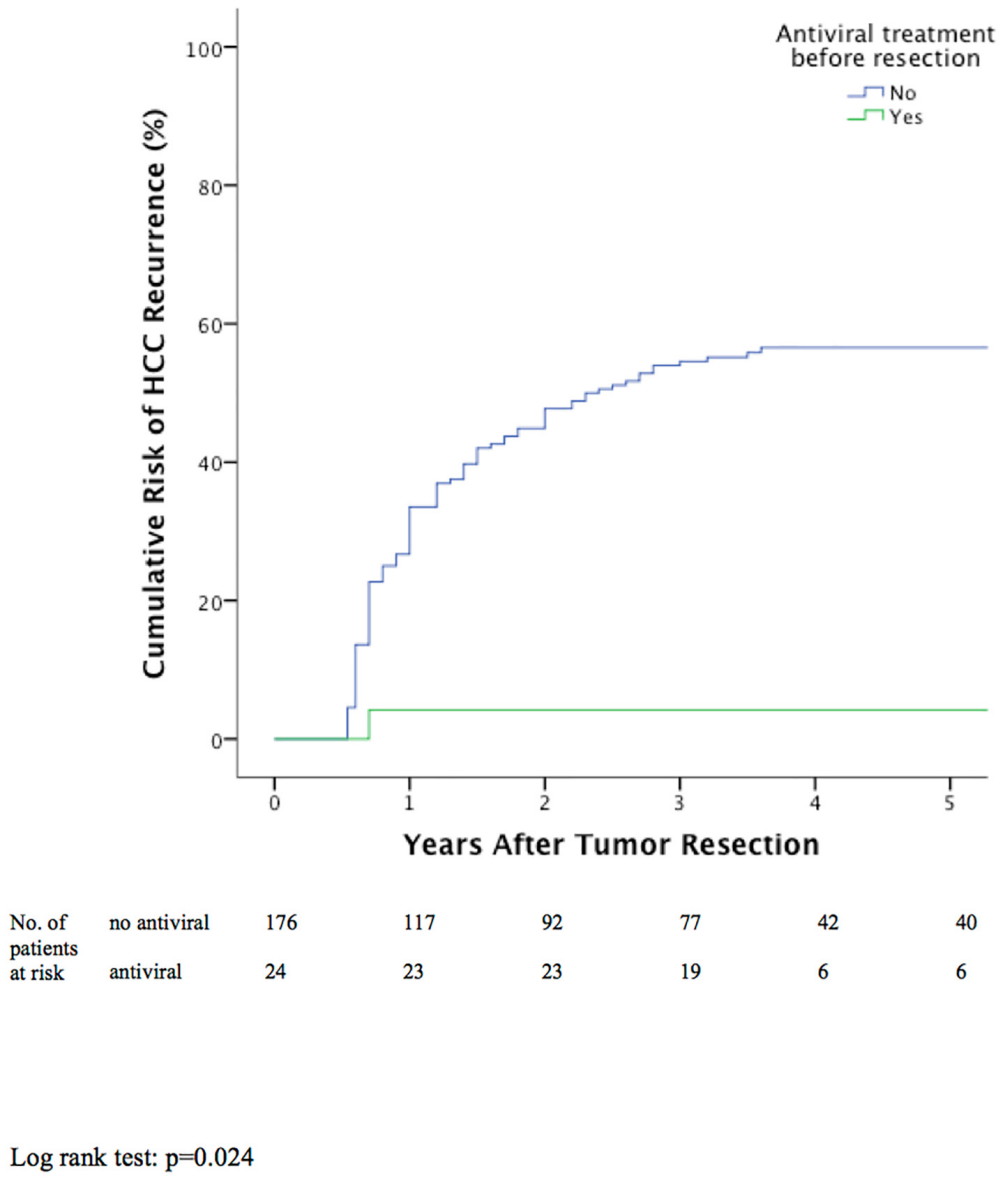
Cumulative tumor recurrence between patients with or without antiviral treatment before resection.

Significant factors associated with higher cumulative risk of tumor recurrence by univariable analyses included AFP >100 ng/mL at the time of resection; tumor size >3cm; poor tumor differentiation; the presence of lymphovascular permeation; the presence of microsatellite lesions; HBV viral load >20,000 IU/mL pre-resection, at 1 and 6 months post resection; the presence of core promoter and C1653T mutations; patients with antiviral treatment before resection (Table 1) were identified. These data were entered into multivariate analysis (Table 2). Stepwise Cox regression analysis identified that one-month post-resection HBV DNA >20,000 IU/mL (p = 0.019; relative risk (RR) 1.67; 95% C.I.: 1.09–2.57) (Fig 1), the presence of lymphovascular permeation (p<0.001; RR 2.69; 95% C.I.: 1.75–4.12) (Fig 2), the presence of microsatellite lesions (p<0.001; RR 2.86; 95% C.I.: 1.82–4.51) (Fig 3), and AFP >100ng/mL before resection (p = 0.021; RR 1.63; 95% C.I.: 1.08–2.47) (Fig 4) were independently associated with HCC recurrence (Table 2). Antiviral treatment before resection (p = 0.024; RR 0.1; 95% C.I.: 0.01–0.74) (Fig 5) was independently associated with reduced risk of HCC recurrence.

**Table 2:** Multivariable analysis of independent factors associated with tumor recurrence.

Variables	Relative cumulative risk (95% Confidence Interval) of hepatocellular carcinoma recurrence	p Value
**Lymphovascular permeation**	2.69 (1.75–4.12)	<0.001
**Presence of microsatellite lesions**	2.86 (1.82–4.51)	<0.001
**One month post resection HBV DNA >20,000 IU/mL**	1.67 (1.09–2.57)	0.019
**Antiviral treatment before resection**	0.10 (0.01–0.74)	0.024
**AFP >100ng/mL before resection**	1.63 (1.08–2.47)	0.021

### Post-resection Independent Predictive Score (PRIPS)

**Fig 6:**
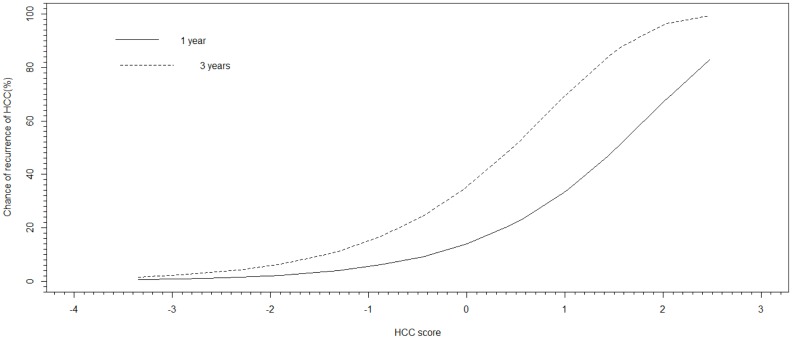
Chance of recurrence of hepatocellular carcinoma at 1 and 3 years according to the different values of the PRIPS.

The derived PRIPS after subtracting the tentative optimal score was formulated as [0.43 * AFP<100ng/mL before resection + 1.01 * presence of lymphovascular permeation + 1.03 * presence of microsatellite lesions + 0.45 * one-month post-resection HBV DNA >20,000 IU/mL—2.90 * antiviral treatment before resection– 0.45, with each variable either as presence = 1 or absence = 0]. The relative risk of the PRIPS for HCC recurrence was 2.71 (95% C.I.: 2.12–3.48, p<0.001). The AUC were 0.675 (95% C.I.: 0.6–0.78) and 0.746 (95% C.I.: 0.69–0.82) for 1- and 3- year prediction respectively. The optimal cut-off of 0 had good sensitivity of 75.3% and 60.6% respectively and specificity of 55.7% and 79.2% respectively, to predict the 1- and 3-year risks for the HCC recurrence after resection (Table 3; Fig 6). In the leave-one-out validation analysis, all accuracy measures highly resembled to those obtained on the total sample, showing good accuracy but with generally wider confidence intervals. The relative risk of the PRIPS by the leave-one-out cross validation was 2.05 (95% C.I.: 1.63–2.57, p<0.001).

**Table 3:** Optimal cut-off values by maximizing Youden index and their accuracies for the PRIPS derived from whole study population and validated with leave-one-out cross-validation.

	1-year prediction		3-year prediction	
	Value	95% CI	Value	95% CI
**Total study population**				
Optimal cut-off	0		0	
Sensitivity	75.3	36.6, 85.5	60.6	46.0, 76.9
Specificity	55.7	46.1, 90.4	79.2	65.2, 76.9
Positive predictive value	34.9	31.0, 59.2	72.1	64.0, 87.0
Negative predictive value	87.7	81.4, 92.3	69.4	64.2, 77.3
Positive likelihood ratio	1.70	1.42, 4.60	2.91	2.01, 7.56
Negative likelihood ratio	0.44	0.27, 0.72	0.50	0.33, 0.63
**Leave-one-out cross-validation**				
Sensitivity	82.5	51.5, 86.3	77.2	55.0, 80.3
Specificity	47.3	47.2, 71.1	55.6	54.2, 83.0
Positive predictive value	33.1	28.4, 42.5	60.7	57.5, 76.8
Negative predictive value	89.5	79.9, 92.6	73.4	63.2, 79.1
Positive likelihood ratio	1.57	1.25, 2.34	1.74	1.53, 3.73
Negative likelihood ratio	0.37	0.25, 0.80	0.41	0.30, 0.66

## Discussion

This is the first prospective study to comprehensively investigate demographic, biochemical, tumor, virological and antiviral treatment for risk factors associated with recurrence of HCC after resection. In this study, the median time to HCC recurrence post-resection was 184 weeks, which is comparable to the 34 months as reported in the study by Shah SA et al [20]. and longer than the 24 and 11.9 months reported in the study by Cha C et al. and Kumar AM et al. respectively [21,22]. The median follow-up time of 207 weeks was also much longer than the 75 weeks in our previous study [13]. We have included additional risk factors namely, HBV DNA level before and after HCC resection, pre-S deletions, C1653T and T1753V mutations, MELD and Child-Pugh scores, and surgical factors including blood loss and anatomical resection in the current study [23]. Baseline demographics including male predominance and median age of diagnosis were similar in both studies. However, age >60 years was no longer associated with recurrence. The ‘age factor’ associated with recurrence after resection remains controversial. A recent study found younger age was associated with more aggressive disease, shorter survival and higher recurrence rate [24], another study suggested otherwise [25].

Biochemical and histopathological are strongly associated with tumor recurrence post-resection. In this study, AFP >100ng/mL before resection and lymphovascular permeation were independently associated with recurrence [13,23]. Similar pathological risk factors predicting recurrence patterns were found in a recent study.[26]. High serum AFP level suggests tumor vascular invasion and was associated with poor recurrence-free survival [27]. Cirrhosis was not found to be associated with recurrence in both of our studies [13]. The cirrhosis factor remained controversial. Early studies have suggested that cirrhosis associated with recurrence [28,29], whereas a more recent study in a Western cohort suggested that non-cirrhotic patients were associated with higher recurrence rate [30]. Biochemical factors including MELD and Child-Pugh scores, surgical factors including blood loss and anatomical resection were not associated with recurrence in our study.

High HBV viral load was found to be strongly associated with recurrence after various interventions including liver resection, transcatheter arterial embolization and radiofrequency ablation [13,31–35]. Ongoing HBV viral replication can induce active hepatitis, oxidative stress, fibrosis and subsequent tumor recurrence. Integration of the oncogenic HBV DNA into the host hepatocytes can alter transforming growth factor-beta1 and alpha (2)-macroglobulin production and inhibit the effect of tumor suppressor genes, resulting in uncontrolled cellular proliferation and malignancy [36]. Patients with high HBV viral load after the resection will therefore be more prone to recurrence. There is also an increased risk of multicentric recurrent tumors in the liver remnant and subsequent recurrence.

Recent meta-analysis demonstrated that the presence of pre-S, C1653T, T1753V, and core promoter mutations are associated with an increased risk of HCC [37]. It is likely that these mutations are associated with enhanced viral replication, resulting in hepatocarcinogenesis. One study even suggested the presence of A1762T/G1764A double mutations might act in synergy with C1653T to increase the risk of HCC in patients with HBV genotype C2 infection [38]. Nevertheless, the presence of genotype C and pre-S deletion were not associated with recurrence in the current study. This concurred with findings from another recent study, which suggested postoperative recurrence, or survival period may not be predominantly affected by the genomic changes [24], but more closely associated with tumor characteristics.

The Cox regression model identified the presence of lymphovascular permeation, the presence of microsatellite lesions, one month post-resection HBV DNA >20,000 IU/mL, no antiviral treatment before resection and AFP >100ng/mL as the 5 independent factors associated with higher cumulative risk of tumor recurrence after resection. Among these 5 risk factors, antiviral treatment before or after resection was the only amenable factor. Results from small studies regarding the effectiveness of nucleoside analogues in HCC recurrence have been controversial [39,40], whereas a large study of by Chan et al. [41], reported that the 5-year tumor free survival rate in antiviral group was significantly higher than the control. More recently a large cohort study from Taiwan [42] demonstrated that the 6-year cumulative recurrence and the overall mortality were significantly lower with nucleoside analogue use. The likely mechanism was that antiviral treatment decreased viral load, thereby reducing the risk of inflammation, fibrosis and subsequently recurrence of the HCC. Another study showed that patients with low or undetectable HBV DNA (<2,000 IU/mL) and not on antiviral agents are still at risk of developing HCC [43]. Therefore, pre-surgery HBV DNA and antiviral agent before surgery remain important factors in predicting HCC recurrence. Antiviral treatment should be given to all patients with low or undetectable HBV DNA level before resection. For patients in resource poor countries, antiviral treatment could be prioritized according to the PRIPS.

The PRIPS was derived from the 5 independent risk factors including viral, pathological and biochemical factors. This allows a more comprehensive assessment compared to other proposed scoring system in predicting HCC recurrence, which either focuses on pathological or biochemical factors. El-Assal ON et al. [44] used an invasiveness score based on 6 pathological parameters, Roayaie S et al. [45] used a system of microvascular invasion, Pan QX et al. [46] used the Glasgow Prognostic Score based on C-reactive protein and albumin level, Yamamura K et al. [47] used the neutrophil to lymphocyte ratio (NLR) prognostic score, Zhao WH et al. [48] used the pre-surgery Cancer of the Liver Italian Program (CLIP) score and Shim JH et al. [49] used the preoperative AFP<20ng/mL to predict HCC recurrence. Only the study by Shim JH et al. provided an AUC analysis of the performance of their AFP model to compare with our PRIPS model. The PRIPS model AUC for the 1- and 3-year predictions were 0.67 and 0.75 respectively, which is better than the AFP model with an AUC of 0.53 and 0.49 respectively. In addition, we have compared the AUC of the PRIPS against the CLIP score using the current cohort. The AUC of the CLIP score for 1- and 3-year prediction were 0.509 and 0.497, which was lower than the AUROC of the PRIPS (AUROC of PRIPS for 1-yr = 0.675 and 3-ys = 0.746; p-value ≤0.001).

This score was validated by the stringent leave-one out statistical analysis with high sensitivity and specificity of 82.5% and 47.3% respectively for the prediction at 1 year. There was a slight change in sensitivity and specificity of 77.2% and 55.6% for the prediction at 3 years. This was the first derived risk score to predict HCC recurrence after resection with integration of viral, biochemical and histopathological factors. Using this novel recurrence risk score, clinicians can predict and subsequently reduce the risk of recurrence by starting patients on antiviral treatment before or even after tumor resection. The estimated risk for 1- and 3-year HCC recurrence in patients with 4 risk factors of lymphovascular permeation, microsatellite lesions, one month post-resection HBV DNA >20,000 IU/mL and AFP >100ng/mL would be 80% and 99% respectively. By commencing antiviral treatment before resection, the 1-year risk would be reduced to 8% and the 3-year risk would be reduced to 24%.

There were several limitations of this study. Firstly, We were unable to perform external validation to compare PRIPS with other prediction models with a different population at this stage. We plan to perform external validation in future prospective study. Secondly, subjects were not entirely prospectively recruited. Thirdly, there has been no accuracy estimation method that can be correct in all situations [50,51]. Leave-one-out is almost unbiased and was chosen to provide accuracy estimates of minimum bias [52], rather than bootstrapping. Bootstrapping has been shown to have large bias in its estimates, as illustrated in some real situations [53]. However, it is an expensive procedure with high sample size requirement. Hence, external validation using independent dataset would be necessary to confirm the accuracy of the proposed risk prediction model.

In conclusion, the present large-scale study had identified several tumor, virological and biochemical factors associated with higher cumulative risks of HCC recurrence after resection. In addition, a novel risk score PRIPS was derived to predict the risk of HCC recurrence after resection. Regardless of the HBV DNA level, antiviral treatment should be given to patients before resection to reduce the risk of recurrence.
